# Long-Term Effects of Ivabradine on Cardiac Vagal Parasympathetic Function in Normal Rats

**DOI:** 10.3389/fphar.2021.596956

**Published:** 2021-04-08

**Authors:** Alina Scridon, Vasile Bogdan Halaţiu, Alkora Ioana Balan, Dan Alexandru Cozac, Valeriu Moldovan, Claudia Bănescu, Marcel Perian, Răzvan Constantin Şerban

**Affiliations:** ^1^ University of Medicine, Pharmacy, Science and Technology “George Emil Palade” of Târgu Mureş, Târgu Mureş, Romania; ^2^ Center for Advanced Medical and Pharmaceutical Research, Târgu Mureş, Romania; ^3^ Emergency Institute for Cardiovascular Diseases and Transplantation Târgu Mureş, Târgu Mureş, Romania

**Keywords:** cardioinhibitory response, HCN4, *I*_f_ current, ivabradine, vagal tone

## Abstract

**Background:** The complex interactions that exist between the pacemaker current, *I*_f_, and the parasympathetic nervous system could significantly influence the course of patients undergoing chronic therapy with the *I*_f_ blocker ivabradine. We thus aimed to assess the effects of chronic ivabradine therapy on autonomic modulation and on the cardiovascular response to *in situ* and *in vitro* parasympathetic stimulation. The right atrial expression of HCN genes, encoding proteins for *I*_f_, was also evaluated.

**Methods:** Sympathetic and parasympathetic heart rate variability parameters and right atrial *HCN*(1-4) RNA levels were analyzed in 6 Control and 10 ivabradine-treated male Wistar rats (IVA; 3 weeks, 10 mg/kg/day). The heart rate (HR) and systolic blood pressure (SBP) responses to *in situ* electrical stimulation of the vagus nerve (2–20 Hz) were assessed in 6 additional Control and 10 IVA rats. The spontaneous sinus node discharge rate (SNDR) response to *in vitro* cholinergic receptors stimulation using carbamylcholine (10^−9^–10^−6^ mol/L) was also assessed in these later rats.

**Results:** Ivabradine significantly increased vagal modulation and shifted the sympatho-vagal balance toward vagal dominance. In Control, *in situ* vagus nerve stimulation induced progressive decrease in both the SBP (*p* = 0.0001) and the HR (*p*< 0.0001). Meanwhile, in IVA, vagal stimulation had no effect on the HR (*p* = 0.16) and induced a significantly lower drop in SBP (*p*< 0.05). IVA also displayed a significantly lower SNDR drop in response to carbamylcholine (*p*< 0.01) and significantly higher right atrial *HCN4* expression (*p* = 0.02).

**Conclusion:** Chronic ivabradine administration enhanced vagal modulation in healthy rats. In addition, ivabradine reduced the HR response to direct muscarinic receptors stimulation, canceled the cardioinhibitory response and blunted the hemodynamic response to *in situ* vagal stimulation. These data bring new insights into the mechanisms of ivabradine-related atrial proarrhythmia and suggest that long-term *I*_f_ blockade may protect against excessive bradycardia induced by acute vagal activation.

## Introduction

The heart rate is the result of a highly coordinated sequence of electrical phenomena that normally take place in the pacemaker cells of the sinus node. The autonomic nervous system (ANS) ensures both long-term, tonic control and short-term, reflex adaptation of the HR to internal and external factors. The hyperpolarization-activated inward current (*I*_f_) is critical in establishing the HR and one of the most relevant targets of HR modulation by the ANS (DiFrancesco, 2010).

Ivabradine is a specific *I*_f_ blocker devoid of dromotropic, inotropic, and lusitropic effects (Camm and Lau, 2003; Manz et al., 2003). In patients with heart failure, chronic ivabradine administration significantly improved clinical outcomes (Swedberg et al., 2010), while also causing a significant, although modest increase in atrial fibrillation, but not in ventricular arrhythmias occurrence (Martin et al., 2014; Cammarano et al., 2016). Studies have also shown that, by inhibiting *I*_f_, ivabradine can significantly reduce the tachycardic response to acute sympathetic stimulation and can thus provide benefit in a wide range of settings associated with sympathetic hyperactivation. In patients with postural orthostatic tachycardia syndrome, ivabradine significantly improved the quality of life (Taub et al., 2021) and efficiently reduced the HR at rest and during tilting (Gee et al., 2018; Taub et al., 2021). In a small randomized controlled trial, ivabradine therapy significantly improved symptoms in patients with inappropriate sinus tachycardia (Cappato et al., 2012). Promising results have also been reported in patients with sinus tachycardia-mediated vaso-vagal syncope, in whom ivabradine was well tolerated and was associated with marked benefit or complete resolution of symptoms (Sutton et al., 2014).

The relationship between *I*_f_ blockade and parasympathetic activation appears to be, however, much more complex. In rats with post-myocardial infarction heart failure, long-term ivabradine therapy was shown to counteract the increase in the expression of genes encoding for hyperpolarization-activated cyclic nucleotide-gated (HCN) channels, responsible for generating *I*_f_, in the ventricular myocardium (Suffredini et al., 2012). Meanwhile, chronic ivabradine administration was associated with a significant increase in sinus node *HCN4* expression in mice (Leoni et al., 2006) If ivabradine exerts such an effect on sinus node cells, this could significantly alter the HR response to vagal stimulation. Acute (Mangin et al., 1998), but not chronic (Silva et al., 2016) intraperitoneal ivabradine administration has also been associated with marked increase in heart rate variability (HRV) parameters in rats. In addition, clinical and experimental data indicate that in the setting of heart failure, long-term ivabradine therapy shifts the sympatho-vagal balance toward vagal dominance (Milliez et al., 2009; Kurtoglu et al., 2014; Böhm et al., 2015). Accumulating data therefore suggest that chronic ivabradine therapy could alter both the vagal modulation and the HR response to vagal stimulation and could thus influence the risk of patients prone to cardiac arrhythmias and the clinical course of patients with vaso-vagal syncope. However, the effects of chronic *I*_f_ blockade on the HR response to acute parasympathetic stimulation have not been evaluated to date and its impact on vagal modulation in settings other than heart failure remains to date unknown.

We therefore aimed to help solving some of the existing controversies and to increase knowledge regarding the effects of the *I*_f_ blocker ivabradine. To achieve this goal, we designed an experimental study to assess the effects of chronic ivabradine therapy on the HR response to *in situ* and *in vitro* acute parasympathetic stimulation. The impact of chronic ivabradine administration on the sympatho-vagal modulation and on the right atrial expression level of *HCN* channels was also evaluated.

## Materials and Methods

### Studied Animals

**FIGURE 1:**
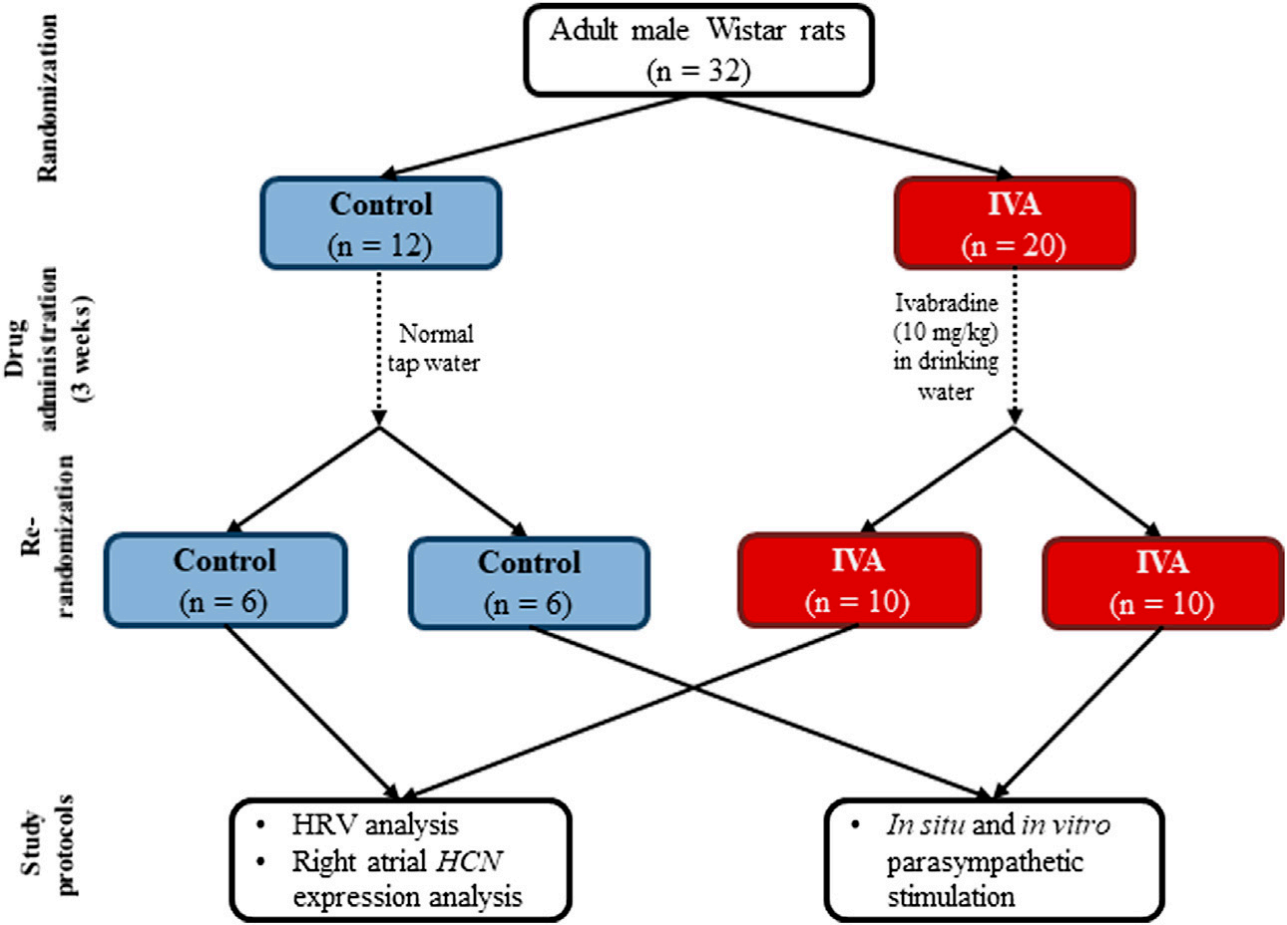
The flow chart of the study design for the Control and the ivabradine-treated (IVA) groups. HRV – heart rate variability

The flow chart of the study design is presented in Figure 1. Adult male Wistar rats (200–250 g) obtained from the local animal facility were initially randomized into two groups: Control (*n* = 12) and IVA (*n* = 20). All animals were housed individually in polycarbonate cages, in a temperature-controlled room (21 –24°C), with a 12/12 h light/dark cycle, and had free access to water and standard food. Rats in the IVA group received a daily dose of 10 mg/kg of body weight of ivabradine (ivabradine hydrochloride; Servier; Courbevoie, France) in their drinking water throughout the study, starting three weeks prior to any experimental procedure. To ensure that each rat consumed the adequate amount of ivabradine, the drug was dissolved in 15 ml of water; as soon as this amount was consumed, normal tap water was put at the rats’ disposal for the rest of the day. Control rats received normal tap water throughout the study. The daily water intake was measured in all rats throughout the study. All protocols complied with the International Council for Laboratory Animal Science guidelines (Directive 2010/63/EU) and were approved by the local Ethics Committee and the National Sanitary Veterinary and Food Safety Authority. Partial blinding was applied in the present study: the researchers who performed the experimental procedures, (i.e., ECG device implantation, vagus nerve stimulation, atrial sampling, *in vitro* studies) were not blinded to the study group, but all parameters were measured and all statistical analyses were performed in a blinded manner.

### Continuous 24 h ECG Monitoring and Heart Rate Variability Analysis

Three weeks after the beginning of the study, Control and IVA rats were re-randomized into two subgroups each. Six Control and 10 IVA rats were implanted with radiotelemetry ECG devices (TA11 CA-F40; Data Sciences International, St. Paul, MN), as described previously (Doñate Puertas et al., 2017). After one week of post-implantation recovery, 24 h continuous ECG monitoring was performed in unrestrained, “conscious” rats, and the mean baseline HR was calculated using a program developed to automatically detect the R waves and measure the RR intervals (Gallet et al., 2013). Heart rate variability analysis was performed based on the 24 h ECG recordings by analyzing beat-to-beat variations in RR intervals in the time and frequency domains, as described previously (Scridon et al., 2012). For the time domain analysis, the standard deviation of normal RR intervals (SDNN), the root mean square of the successive RR-interval differences (RMSSD), and the percentage of successive RR intervals that differed by > 5 ms (pNN5) were assessed. For the frequency domain analysis, time series were resampled at 20 Hz using a cubic spline, then spectral power in the low-frequency (LF) band (0.3–0.6 Hz), the high-frequency (HF) band (0.6–2.5 Hz), and the LF/HF ratio were estimated on 2,048-point (102.4 s) segments windowed by the Hanning function and overlapping by 50% using a fast Fourier transform. All ECG tracings were assessed visually and all artifacts, arrhythmic events, and compensatory pauses were excluded prior to HRV analysis.

### Right Atrial Expression Analysis of Genes Encoding for Hyperpolarization-Activated Cyclic Nucleotide-Gated Channels

At the end of the ECG monitoring period, the 6 Control and 10 IVA rats were euthanized using an intraperitoneal injection of a terminal dose of sodium pentobarbital (>100 mg/kg). The thoracic cavity was opened and the heart was removed. The free wall of the right atrium was collected and rapidly immersed into RNA stabilization solution (RNAlater; Thermo Fisher Scientific, Waltham, MA). The RNA was isolated using iPrep PureLink Total RNA Kits and the iPrep Purification Instrument (Thermo Fisher Scientific). Reverse transcription was performed using the SuperScript VILO cDNA Synthesis Kit (Thermo Fisher Scientific). The RNA expression levels of three target genes, (i.e. *HCN1*, *HCN2*, and *HCN4*) and one control gene, (i.e. glyceraldehyde 3-phosphate dehydrogenase [*GAPDH*]) were analyzed using a customized fast 96-well plate containing TaqMan Gene Expression Assays for the tested genes (Thermo Fisher Scientific). The expression of the neuron-specific *HCN3* isoform was also analyzed. All experiments were performed on a 7500 Fast Dx Real-Time PCR System (Applied Biosystems, Waltham, MA). The expression levels of *HCN1*, *HCN2*, *HCN3*, and *HCN4* were normalized with *GAPDH* housekeeping gene levels and compared between the two groups.

### *In situ* and *in vitro* Parasympathetic Stimulation

Three weeks after the beginning of the study, the remaining 6 Control and 10 IVA rats were submitted to vagus nerve stimulation under ketamine/medetomidine anesthesia (i.p., 75.0/0.5 mg/kg). Briefly, the anterior cervical region was dissected and the sternohyoid and sternocleidomastoid muscles were separated and retracted laterally to allow visualization of the carotid artery. The right vagus nerve was carefully isolated from the surrounding connective tissue. In order to avoid interferences from retrograde electrical vagus nerve stimulation, the nerve was secured using two surgical threads and was then cut. A stimulation electrode was placed beneath the distal end of the vagus nerve and was elevated to avoid contact with the surrounding tissues. The nerve was then stimulated electrically using rectangular impulses (pulse duration 0.5 ms; 20 V) at progressively higher frequencies, (i.e. 2, 5, 10, and 20 Hz). Each stimulation protocol was applied for 15 s, with 5 min intervals between stimulations. Surface ECG was continuously recorded during the entire duration of the protocol and the HR was calculated at baseline and during each stimulation protocol based on RR intervals duration. The ECG signal was captured using three electrodes placed on the two upper limbs and on the left lower limb, was amplified, and delivered to the acquisition board. The ECG signal was recorded using an acquisition program developed using the LabVIEW 8.20 software (National Instruments, Austin, TX). Systolic blood pressure (SBP) was measured non-invasively at baseline and during each stimulation protocol using a photoplethysmographic method, as described previously (Scridon et al., 2015). Briefly, a pneumatic tail cuff was placed proximally on the rat’s tail and inflated/deflated using the PE-300 programmed electro-sphygmomanometer (Narco Bio-Systems Inc., Houston, TX). The photoplestimography sensor was placed on the tail distally to the pneumatic cuff, with the infrared beam at the level of the caudal artery. The cuff pressure and the phototransducer signals were routed to the signal acquisition board. The signals were recorded using an acquisition program developed using the LabVIEW 8.20 software (National Instruments).

At the end of the stimulation protocols, the anesthetized rats were euthanized by thoracotomy. The hearts were explanted and rapidly immersed into prewarmed (37°C) oxygenated (95% O_2_; 5% CO_2_) Krebs-Henseleit solution containing NaCl (118.00 mM), KCl (4.70 mM), NaHCO_3_ (25.00 mM), MgSO_4_ (1.20 mM), CaCl_2_ (1.25 mM), KH_2_PO_4_ (1.20 mM), and glucose (11.00 mM). The right atrium was isolated and transferred into the Steiert organ bath (Hugo Sachs Elektronik-Harvard Apparatus; March-Hugstetten, Germany) containing oxygenated Krebs-Henseleit solution at 37°C. The spontaneous sinus node discharge rate was measured at baseline and after direct cholinergic receptors stimulation using carbamylcholine solutions with progressively higher concentrations (10^−9^ mol/L to 10^−6^ mol/L, prewarmed at 37°C). Each solution was applied for 10 min. All samples were washed for 5 min with oxygenated Krebs-Henseleit solution between exposures to the successive carbamylcholine solutions.

### Statistical Analysis

Statistical analyses were undertaken using MedCalc for Windows, version 12.4.3.0 (MedCalc Software; Ostend, Belgium). A two-tailed *p*-value < 0.05 was considered statistically significant. All data were tested for normality and are expressed as means ± standard error of the mean or median and interquartile range, as appropriate. Between-group comparisons were performed using the unpaired Student’s *t*-test or the Mann–Whitney *U*-test, as appropriate. Differences within the same group were tested for significance using the paired Student’s *t*-test or the Wilcoxon matched-pairs signed-rank test, as appropriate, and repeated-measures ANOVA. Changes in the spontaneous sinus node discharge rate in response to carbamylcholine administration were also analyzed by a nonparametric two-way ANOVA, factoring for the effects of ivabradine treatment status (ivabradine-treated vs. non-treated) and carbamylcholine concentrations (10^−9^ mol/L–10^−6^ mol/L). Due to the very limited amount of data available on this topic, the sample size could not be calculated prior to the study. However, based on previous studies in non-treated rats, Control rats were expected to present low interindividual variability and a sample size of 6 was therefore considered sufficient for these groups. To compensate for potentially higher interindividual differences in the ivabradine-treated animals, the two IVA groups were designed larger (*n* = 10) than the Control groups.

## Results

There was no significant difference in the daily water intake between the IVA and the Control rats (53.21 ± 2.49 ml/24 h vs. 57.50 ± 3.30 ml/24 h; *p* = 0.34).

### Chronic Ivabradine Administration Increases Vagal Modulation and Shifts the Sympatho-Vagal Balance Toward Vagal Dominance in Healthy Rats

**FIGURE 2:**
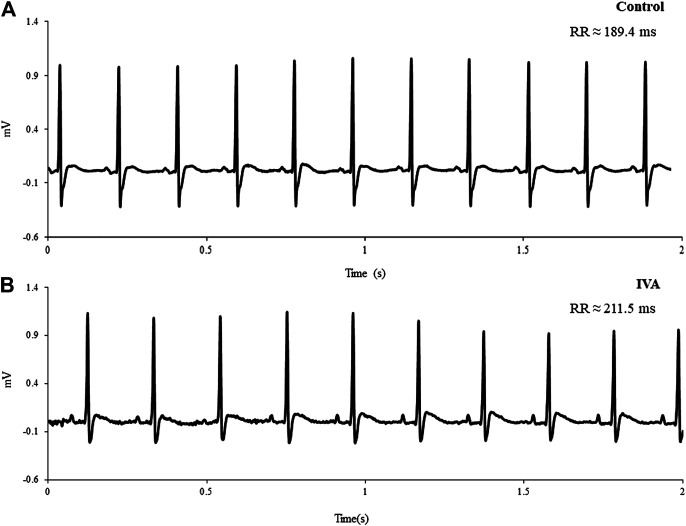
Representative telemetric ECG tracings recorded in a Control **(A)** and an ivabradine-treated (IVA) rat **(B)**

Figure 2 depicts typical telemetric ECG tracings recorded in Control and IVA rats. As expected, mean 24 h HR was significantly lower in the ivabradine-treated rats compared to their non-treated counterparts (*p*< 0.01; Table 1). Mean awake and mean asleep HR were both significantly lower in the ivabradine-treated compared to the non-treated rats (both *p*< 0.0001; Table 1). In the Control rats, there was a 22.6 ± 2.8 bpm (*p*< 0.001) awake/asleep HR difference, compared with an 18.7 ± 5.9 bpm (*p* = 0.01) awake/asleep HR difference in the ivabradine-treated rats (*p* = 0.63).

**TABLE 1:** Mean 24 h heart rate and heart rate variability parameters in ivabradine-treated (IVA) and non-treated (Control) rats.

Parameter	Control (*n* = 6)	IVA (*n* = 10)	*p*-value
*Heart rate (HR)*			
Mean 24 h HR (bpm)	341.5 ± 8.3	301.3 ± 7.5	<0.01
Mean HR awake (bpm)	356.6 ± 5.5	304.6 ± 6.9	<0.0001
Mean HR asleep (bpm)	334.0 ± 6.4	285.9 ± 4.9	<0.0001
24 h *Heart rate variability analysis*			
*Time domain*			
SDNN (ms)	23.3 ± 1.9	26.6 ± 1.7	0.23
RMSSD (ms)	3.9 ± 0.3	5.5 ± 0.2	<0.01
pNN5 (%)	16.9 ± 2.6	28.1 ± 1.7	<0.01
*Frequency domain*			
LF (ms^2^)	1.9 ± 0.2	2.5 ± 0.2	0.11
HF (ms^2^)	6.8 ± 1.5	11.6 ± 1.2	0.03
LF (n.u.)	6.1 ± 0.9	6.1 ± 0.4	1.00
HF (n.u.)	19.8 ± 3.4	32.1 ± 4.2	0.04
LF/HF	0.30 ± 0.02	0.23 ± 0.02	0.04
*Heart rate variability analysis* – *awake*			
*Time domain*			
SDNN (ms)	21.0 ± 1.4	23.8 ± 1.7	0.25
RMSSD (ms)	3.8 ± 0.3	5.3 ± 0.3	<0.01
pNN5 (%)	15.3 ± 2.9	26.1 ± 2.0	<0.001
*Frequency domain*			
LF (ms^2^)	2.2 ± 0.2	2.7 ± 0.1	0.43
HF (ms^2^)	6.2 ± 1.4	11.3 ± 1.3	<0.01
LF (n.u.)	6.4 ± 0.8	6.6 ± 0.4	0.46
HF (n.u.)	18.5 ± 3.7	31.9 ± 4.3	0.04
LF/HF	0.30 ± 0.03	0.21 ± 0.02	0.04
*Heart rate variability analysis* – *asleep*			
*Time domain*			
SDNN (ms)	22.7 ± 1.7	26.4 ± 1.4	0.14
RMSSD (ms)	4.2 ± 0.3	6.0 ± 0.3	<0.01
pNN5 (%)	18.7 ± 2.5	31.8 ± 2.3	<0.01
*Frequency domain*			
LF (ms^2^)	1.6 ± 0.2	2.2 ± 0.2	0.42
HF (ms^2^)	7.4 ± 1.6	13.5 ± 1.6	0.03
LF (n.u.)	5.9 ± 1.0	6.2 ± 0.4	0.79
HF (n.u.)	21.2 ± 4.2	34.6 ± 4.2	0.04
LF/HF	0.32 ± 0.03	0.23 ± 0.03	0.04

The values are expressed as means ± standard error of the mean; *p*-values refer to between-group comparisons based on the unpaired Student’s *t*-test.

HF – high-frequency (0.6–2.5 Hz) signals; HR – heart rate; LF – low-frequency (0.3–0.6 Hz) signals; LF/HF – the ratio of low-to high-frequency components; n.u. – normalized units; pNN5 – percentage of successive RR intervals that differed by > 5 ms; RMSSD – root mean square of successive RR-interval differences; SDNN – standard deviation of normal RR intervals.

Similarly to what was previously reported in the setting of heart failure (Milliez et al., 2009; Kurtoglu et al., 2014; Böhm et al., 2015), HRV analysis revealed a significant increase in vagal modulation and a shift of the sympatho-vagal balance toward vagal dominance in ivabradine-treated healthy rats (Table 1). The RMSSD, pNN5, and the HF components of the HRV spectrum, reflecting vagal modulation, were all significantly higher in the IVA compared with the Control rats (awake, asleep, and over 24 h; all *p* < 0.05; Table 1). The LF/HF ratio, an index of sympathetic and parasympathetic interactions, was significantly lower in the IVA than in the Control rats (awake, asleep, and over 24 h; all *p* < 0.05; Table 1), demonstrating a shift of the sympatho-vagal balance toward vagal dominance in the ivabradine-treated rats. No significant change was observed in the LF components of the HRV spectrum (awake, asleep, and over 24 h; all *p* = NS; Table 1).

### Chronic Ivabradine Administration Up-Regulates the Right Atrial Expression of the Hyperpolarization-Activated Cyclic Nucleotide-Gated Channel 4

**FIGURE 3:**
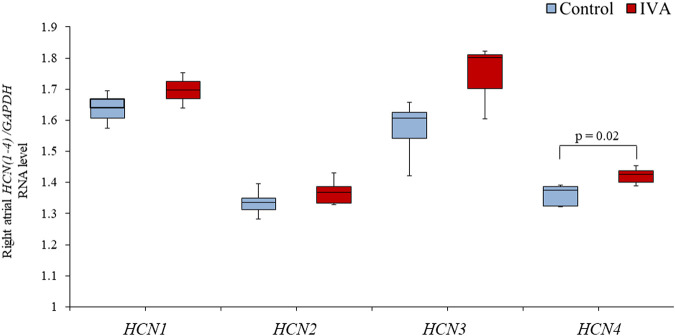
Right atrial RNA expression of genes encoding for hyperpolarization-activated cyclic nucleotide-gated (HCN) channels in the ivabradine-treated (IVA; *n* = 10) and non-treated (Control; *n* = 6) rats. Data are expressed as median and interquartile range (IQR). The ends of the whiskers represent the lowest value within 1.5 IQR of the first quartile and the highest value within 1.5 IQR of the third quartile.

Right atrial *HCN1* and *HCN2* expression levels were similar (both *p* = NS) between the IVA and the Control rats (Figure 3). There was also no significant difference in the right atrial expression of the neuron-specific *HCN3* isoform between the two groups (*p* = 0.22; Figure 3). However, the right atrial expression of *HCN4*, the most highly expressed *HCN* isoform in the sinus node (Sartiani et al., 2017), was significantly higher in the ivabradine-treated compared to the non-treated rats (*p* = 0.02; Figure 3).

### Long-term Ivabradine Administration Cancels the Heart Rate Response and Reduces the Blood Pressure Response to Acute *in situ* Vagal Stimulation

As expected, baseline HR (derived from surface ECG recordings performed in anesthetized rats prior to vagus nerve sectioning) was significantly lower in the IVA compared with the Control rats (202.2 ± 6.6 bpm vs. 246.3 ± 13.6 bpm; *p* = 0.01), whereas SBP was similar between the two groups (143.5 ± 6.9 mmHg vs. 154.1 ± 9.8 mmHg; *p* = 0.58).

**FIGURE 4:**
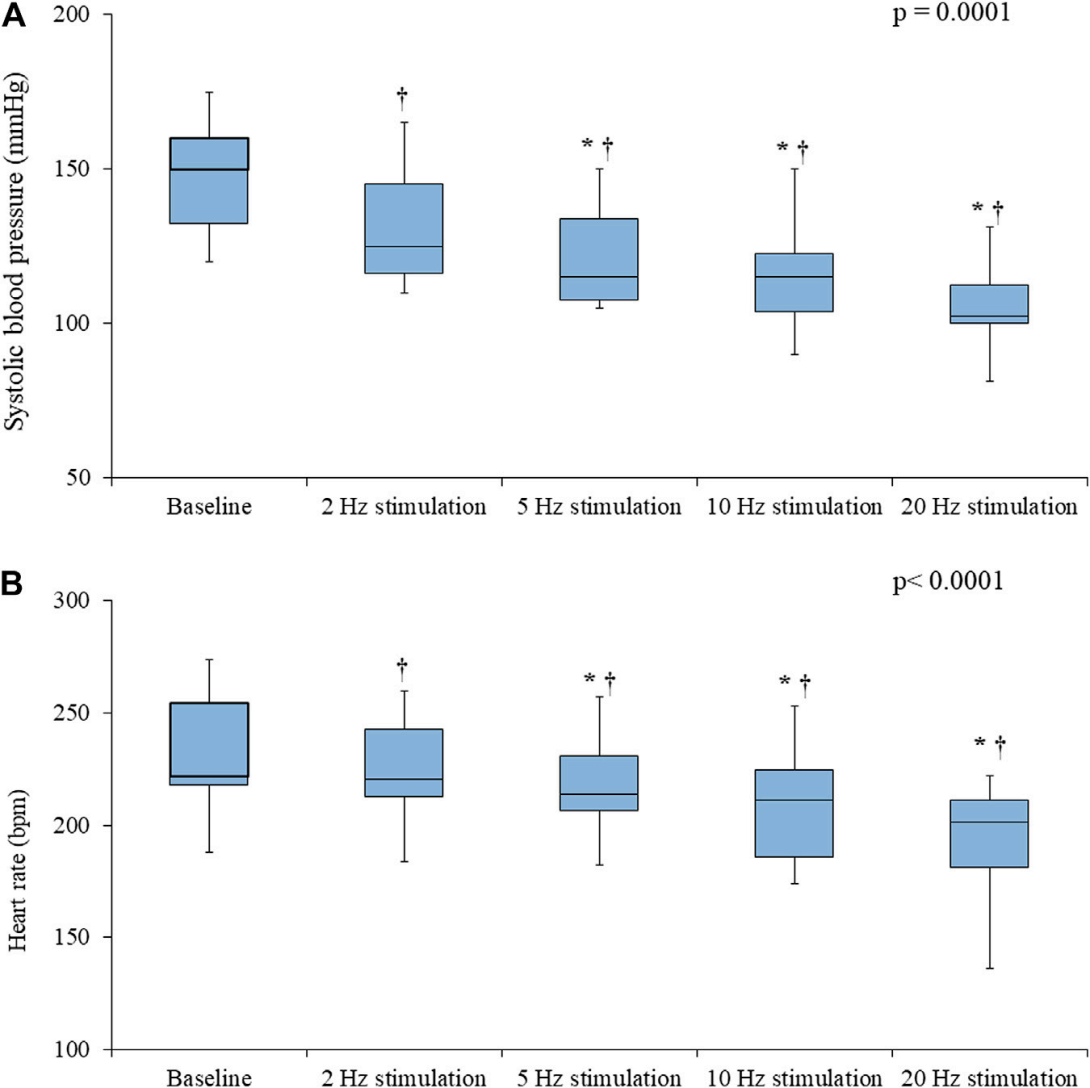
Systolic blood pressure (**A**) and heart rate (**B**) values prior to (baseline) and during vagus nerve stimulation at 2, 5, 10, and 20 Hz in the Control rats (*n* = 6). Data are expressed as median and interquartile range (IQR). The ends of the whiskers represent the lowest value within 1.5 IQR of the first quartile and the highest value within 1.5 IQR of the third quartile. *p*-values in the right upper corners were obtained using nonparametric repeated-measures ANOVA (Friedman test). *p < 0.05 vs. the value obtained during the previous stimulation using the paired Student’s *t*-test or the Wilcoxon matched-pairs signed-rank test, as appropriate; †p < 0.05 vs. baseline using the paired Student’s *t*-test or the Wilcoxon matched-pairs signed-rank test, as appropriate.

**FIGURE 5:**
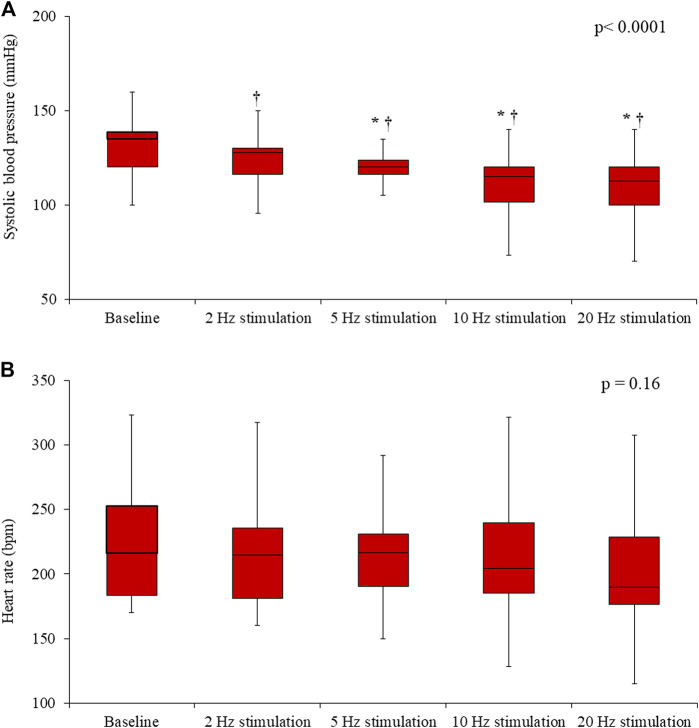
Systolic blood pressure **(A)** and heart rate **(B)** values prior to (baseline) and during vagus nerve stimulation at 2, 5, 10, and 20 Hz in the ivabradine-treated rats (*n* = 10). Data are expressed as median and interquartile range (IQR). The ends of the whiskers represent the lowest value within 1.5 IQR of the first quartile and the highest value within 1.5 IQR of the third quartile. *p*-values in the right upper corners were obtained using nonparametric repeated-measures ANOVA (Friedman test). *p < 0.05 vs. the value obtained during the previous stimulation using the paired Student’s *t*-test or the Wilcoxon matched-pairs signed-rank test, as appropriate; †*p* < 0.05 vs. baseline using the paired Student’s *t*-test or the Wilcoxon matched-pairs signed-rank test, as appropriate.

**FIGURE 6:**
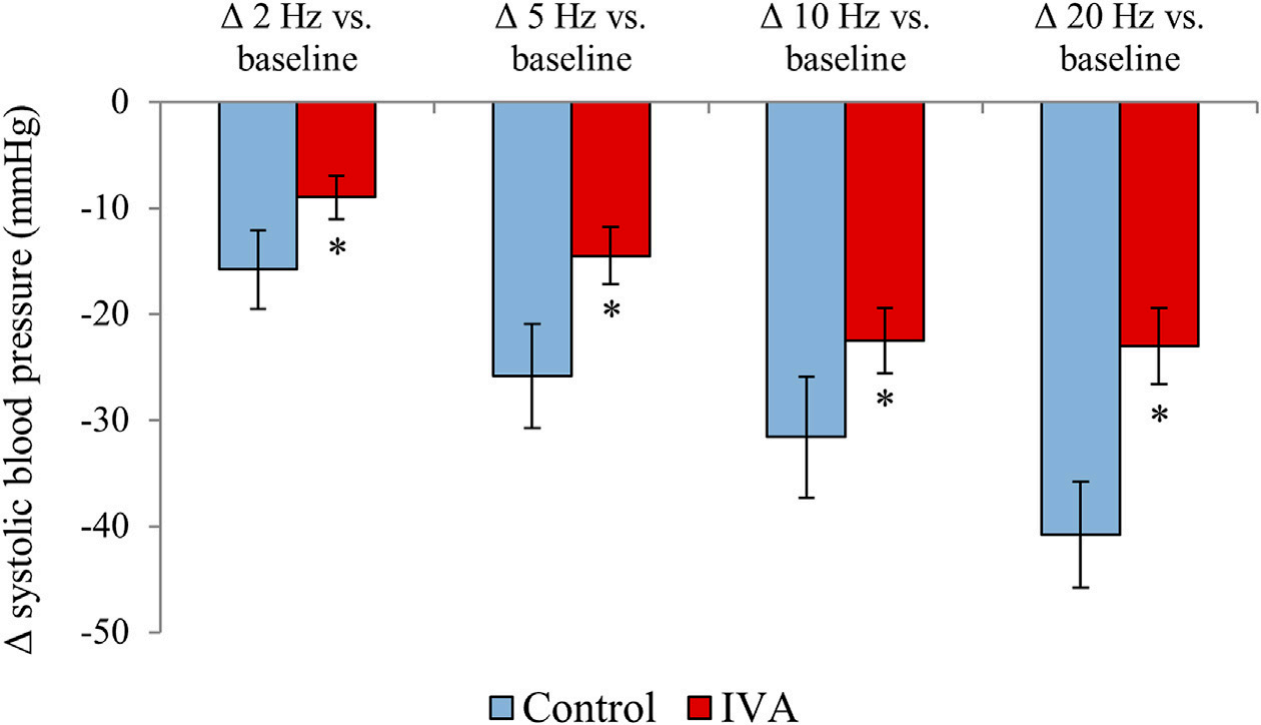
Systolic blood pressure changes during vagus nerve stimulation at 2, 5, 10, and 20 Hz compared with the baseline values in the ivabradine-treated (IVA; *n* = 10) and non-treated (Control; *n* = 6) rats. Data are expressed as means ± standard error of the mean. *p < 0.05 for IVA vs. Control rats using the paired Student’s *t*-test or the Mann-Whitney *U* test, as appropriate.

In the Control rats, electrical stimulation of the vagus nerve induced a significant, progressive decrease in both the SBP (*p* = 0.0001) and the HR (*p* < 0.0001) (Figure 4). In the IVA rats, electrical stimulation of the vagus nerve also induced a significant, progressive decrease in the SBP (*p* < 0.0001) (Figure 5). However, in the ivabradine-treated rats, vagus nerve stimulation had no effect on the HR (*p* = 0.16; Figure 5). In addition, the decrease in SBP in response to vagus nerve stimulation was also significantly lower in the IVA compared with the Control rats for all four stages of the stimulation protocol (all *p* < 0.05; Figure 6).

### Ivabradine Reduces the Drop in the Sinus Node Discharge Rate in Response to *in vitro* Cholinergic Receptors Stimulation

**FIGURE 7:**
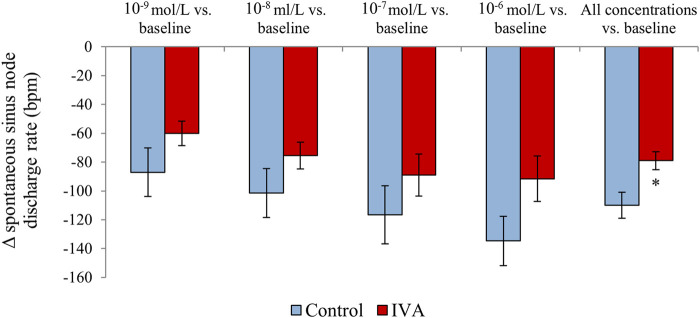
Spontaneous sinus node discharge rate changes in response to *in vitro* carbamylcholine administration (10^−9^ to 10^−6^ mol/L) compared with the baseline values in the ivabradine-treated (IVA; *n* = 10) and non-treated (Control; *n* = 6) rats.

When right atrial samples were assessed *in vitro* under progressively higher carbamylcholine concentrations, both IVA and Control rats displayed a significant, progressive decrease in the spontaneous discharge rate of the sinus node (both *p*< 0.0001; Figure 7). There was no significant difference in the sinus node discharge rate drop in response to similar carbamylcholine concentrations between the two groups (*p* = NS for all carbamylcholine concentrations; Figure 7). However, in absolute value, the drop in the sinus node discharge rate was ∼30–40 bpm lower in the ivabradine-treated than in the non-treated rats for all carbamylcholine concentrations (Figure 7). Moreover, when the responses of the sinus node discharge rate to all carbamylcholine concentrations were combined, the decrease in the spontaneous discharge rate of the sinus node in response to carbamylcholine administration was significantly lower in the IVA compared with the Control rats (*p* < 0.01; Figure 7). In addition, two-way ANOVA factoring for the effects of ivabradine treatment status (ivabradine-treated vs. non-treated) and carbamylcholine concentrations (10^−9^ mol/L–10^−6^ mol/L) demonstrated that the response of the spontaneous discharge rate of the sinus node to parasympathetic stimulation was significantly affected by both carbamylcholine concentration (*p* = 0.04) and ivabradine administration (*p* < 0.01), and that there was no interaction between the two factors (*p* = 0.93).

## Discussion

The ANS is one of the most important contributors to HR regulation, and, although several subcellular components contribute to HR regulation by the ANS, including the acetylcholine-dependent potassium current, calcium currents, and the Na^+^/Ca^2+^ exchanger, much of this regulation is achieved *via I*_f_ modulation (DiFrancesco, 2010). The ANS is, however, a double path (afferent and efferent) neural system and any significant change in cardiovascular parameters is naturally followed by adaptive changes in ANS functioning. The autonomic control of *I*_f_ and the molecular mechanisms underlying *I*_f_ modulation by the sympathetic and the parasympathetic nervous systems are well understood (DiFrancesco, 2010). Conversely, the effects of chronic *I*_f_ blockade on the ANS and on the HR response to acute autonomic changes are still largely unknown. Our study demonstrates that chronic ivabradine administration enhances vagal modulation and shifts the autonomic balance toward vagal dominance in healthy rats. Moreover, we show that chronic *I*_f_ blockade using ivabradine increases right atrial *HCN4* expression, reduces the HR response to direct muscarinic receptors stimulation, cancels the cardioinhibitory response and blunts the hemodynamic response to *in situ* vagal stimulation. These data bring new insights into the mechanisms of ivabradine-related atrial proarrhythmia and provide evidence that long-term *I*_f_ blockade may protect against excessive bradycardia induced by acute vagal activation.

### Chronic Ivabradine Administration Increases Vagal Modulation and Shifts the Autonomic Balance Toward Vagal Dominance. Potential Implications for Ivabradine-Related Atrial Proarrhythmia

Autonomic imbalance with sympathetic hyperactivity is a common denominator of a wide variety of cardiovascular diseases (Brook and Julius, 2000; Hellstrom, 2007; Floras and Ponikowski, 2015). In settings such as heart failure, sympathetic hyperactivity develops as an adaptive mechanism aiming to preserve the cardiac output (Floras and Ponikowski, 2015). However, over the long term, this increased sympathetic activity significantly contributes to impaired prognosis and increased mortality rates (Floras, 2009; Jacobson et al., 2010; Fallavollita et al., 2014). Meanwhile, strategies aiming to decrease the sympathetic and/or increase the parasympathetic tone are believed to have great potential (van Bilsen et al., 2017), and *beta*-blockers, which decrease the cardiac effects of sympathetic activation and circulating catecholamine, have already been shown to increase survival and improve prognosis in heart failure (Hjalmarson et al., 1999; Lechat et al., 1999; Flather et al., 2005).

Accumulating data suggest that “pure” HR-lowering agents could provide similar benefits (Swedberg et al., 2010) and clinical and experimental studies suggest that this beneficial effect could be at least partly due to the ability of *I*_f_ blockers to improve HRV parameters and to induce sustained increase in vagal tone (Mangin et al., 1998; Milliez et al., 2009; Kurtoglu et al., 2014; Böhm et al., 2015; El-Naggar et al., 2018), although this was not the case in the study by Silva et al. (Silva et al., 2016). However, in this latter study, ivabradine was administered intraperitoneally, for only 7–8 days, and the magnitude of HR reduction induced by ivabradine was significantly greater (28%) than that achieved in the present study (13.3%) and in the large clinical trials (15–20%) (Swedberg et al., 2010). This increased drop in HR was also translated into a significant decrease in SBP in the ivabradine-treated rats (Silva et al., 2016), effect that is not encountered at clinically relevant ivabradine doses (Swedberg et al., 2010). Although the exact mechanisms that underlie this effect remain to date unknown, the increase in vagal modulation induced by *I*_f_ blockade could be related to the lengthening of the diastolic filling time and to the consequent ventricular stretch, leading to mechanoreceptors stimulation and thus increasing the vagal and decreasing the sympathetic tone (Oren et al., 1993).

Given the well-known association between heart failure and sympathetic hyperactivity, this shift in autonomic balance toward vagal dominance was interpreted in the previous studies as an ivabradine-induced “improvement” in sympatho-vagal balance (Kurtoglu et al., 2014; Böhm et al., 2015). Although this may be true in settings associated with sympathetic hyperactivity, our data show that chronic ivabradine administration in clinically relevant doses exerts similar effects in healthy subjects. In the present study, chronic ivabradine therapy significantly reduced the HR in 24 h, awake, and asleep recordings, without causing significant dampening of circadian variations in HR. In addition, chronic ivabradine therapy significantly increased vagal modulation (as reflected by the higher parasympathetic indexes RMSSD and pNN5, and the increased HF components of the HRV spectrum), and shifted the sympatho-vagal balance toward vagal dominance (as reflected by the significantly lower LF/HF ratio) in healthy rats. These changes were present both when the animals were awake and while asleep. This demonstrates that the increase in vagal modulation induced by long-term *I*_f_ blockade is independent on the baseline status of the autonomic balance and is not restricted to settings associated with sympathetic hyperactivity. The autonomic changes induced by ivabradine cannot be therefore interpreted as “corrective”; rather, vagal hyperactivity appears to be a common ivabradine “side effect.”

#### Clinical Implications

On the one hand, the fact that the increase in vagal modulation induced by ivabradine is not restricted to the heart failure setting suggests that the beneficial effects of ivabradine could extend to other settings associated with increased sympathetic activity, in which the ivabradine-induced increase in vagal modulation could reduce myocardial oxygen demand and ischemia, diminish sympathetic stimulation and adrenoreceptor-mediated cytotoxicity, apoptosis, and hypertrophy, and reduce the likelihood of ventricular tachyarrhythmias and sudden death (Böhm et al., 2015). On the other hand, vagal hyperactivity has been shown to promote ectopic activity, reentry, and atrial fibrillation *via* multiple mechanisms (Scridon et al., 2018). Given the highly proarrhythmic effects of vagal activation at the atrial level, the increase in vagal modulation induced by ivabradine demonstrated in the present study could provide an explanation for the increased risk of atrial fibrillation associated with ivabradine use in clinical trials (Martin et al., 2014; Cammarano et al., 2016).

### Chronic Ivabradine Administration Cancels the Cardioinhibitory Response and Blunts the Hemodynamic Response to Acute Vagal Stimulation. Potential Implications for Vaso-Vagal Syncope

According to estimates, 40% of individuals experience at least one episode of syncope during their lifetime (Colman et al., 2004), with vaso-vagal syncope representing up to 60% of all syncope cases (Brignole et al., 2006). Yet, the therapeutic management of vaso-vagal syncope remains highly challenging.

In the present study, *in situ* preganglionic vagus nerve stimulation elicited significant arterial hypotension and bradycardia in control rats. However, in the ivabradine-treated rats, vagal stimulation was not followed by the typical decrease in HR and the drop in SBP was significantly lower than that recorded in the control rats. Thus, the present study demonstrates for the first time that chronic ivabradine administration in clinically relevant doses cancels the cardioinhibitory response and blunts the hemodynamic response to acute vagal stimulation in rats. Interestingly, reduced mean blood pressure response and almost complete elimination of HR response to vagus nerve stimulation was also reported following metoprolol administration (Gierthmuehlen and Plachta, 2016). Meanwhile, in apparent contradiction with our data, a higher drop in HR induced by vagus nerve stimulation was reported following ivabradine administration in two recent studies (Uemura et al., 2017; Kawada et al., 2019). Although the use of different anesthetic drugs may account for some of this discrepancy, in both those studies, acute ivabradine administration was used and the drug was administered in a single, intravenous dose. Chronic and acute ivabradine administration therefore appear to exhibit discordant effects on the HR response to vagal stimulation, suggesting that chronic ivabradine therapy could induce remodeling of one or several components of the parasympathetic nervous system-cholinergic receptors-*I*_f_ axis over the long term.

Head-to-head comparisons of sinus node discharge rate responses to direct cholinergic receptors stimulation for each carbamylcholine concentration did not show significant differences between the two groups. However, when the responses to all carbamylcholine concentrations were considered together, the decrease in the spontaneous discharge rate of the sinus node was significantly lower in the ivabradine-treated compared with the control rats. Two-factor ANOVA also demonstrated that the response of the spontaneous discharge rate of the sinus node to parasympathetic stimulation was significantly affected by ivabradine administration. Thus, the cancellation of the HR response to vagal stimulation induced by ivabradine appears to result from local, receptor and/or post-receptor ivabradine-parasympathetic nervous system interferences. In the same vein, our transcriptomic data showed that chronic ivabradine administration significantly up-regulates right atrial *HCN4* expression. In previous studies, ivabradine administration counteracted the increase in ventricular *HCN4* expression in post-myocardial infarction rats (Suffredini et al., 2012). Similarly, ivabradine has been shown to decrease *HCN2* and *HCN4* expression in a mixture of right and left atrial myocytes obtained from transgenic mice overexpressing the (pro)renin receptor (Wang et al., 2019). However, in the study by Leoni et al., 3 weeks ivabradine administration in mice led to a significant increase in sinus node *HCN4* expression (Leoni et al., 2006). The right atrial samples examined in our study contained both nodal and non-nodal tissue. However, since *HCN4* is highly expressed in the nodal pacemaker cells and only sparsely present in the remaining atrial myocardium (Chandler et al., 2009; Li et al., 2014), it is likely that the *HCN4* changes observed in our study reflect sinus node, rather than non-nodal alterations. Thus, in line with the data reported by Leoni et al. (Leoni et al., 2006), our data indicate that, contrary to its effects on non-pacemaker cells, long-term ivabradine therapy augments *HCN4* expression at the level of the sinus node. Eventually, *HCN4* and the consequent *I*_f_ up-regulation could render the sinus node less sensitive to acute vagal inputs and protect against excessive vagal-induced bradycardia. The recent finding of Kozasa et al. that *HCN4* overexpression attenuates the bradycardic response to vagus nerve stimulation (Kozasa et al., 2018) strongly supports this hypothesis.

Ivabradine does not pass the brain-blood barrier (Postea and Biel, 2011) and our data showed that long-term ivabradine therapy does not affect the expression of the neuronal specific *HCN*3 channel, at least at the level of the heart. However, *HCN* channels are also expressed in peripheral autonomic and somatosensory neurons, where they are responsible for generating the neuronal homologous of *I*_f_ – *I*_h_, on which ivabradine has also been shown to exhibit significant inhibition (Savelieva and Camm, 2006; León-Hernández et al., 2016). An inhibiting effect of ivabradine on vagus nerve *I*_h_ activity could therefore also contribute to the lack of the HR response to *in situ* vagal stimulation observed in the ivabradine-treated rats.

#### Clinical Implications

The present study indicates for the first time that ivabradine is not only safe, but is also highly effective in preventing exaggerated vagal-induced bradycardia in rats. These data serve as a basis for future studies that will have to assess the validity of these findings in humans. If confirmed in clinical settings, this protective effect of ivabradine may place ivabradine as a drug that should not only not be avoided in patients with cardioinhibitory vaso-vagal syncope, but could even protect these patients from exaggerated vagal-induced cardioinhibitory response and reduce the risk of vaso-vagal syncope.

### Strengths and Limitations

In the present study, the effects of long-term *I*_f_ blockade on autonomic function and on the cardiovascular response to parasympathetic stimulation were assessed by the use of clinically relevant ivabradine dose and route of administration. Both *in situ* and *in vitro* studies were performed, providing a comprehensive view on the impact of ivabradine therapy on the cardiovascular response to parasympathetic activation. The present study indicates ivabradine-induced sinus node *HCN4* up-regulation as potential mechanism for the blunted cardiovascular response to vagal stimulation seen in these rats. However, further studies are needed to explore other potential mechanisms responsible for this effect. *HCN* expression was not specifically assessed at the level of the sinus node; RNA levels were analyzed using a mixture of nodal and non-nodal right atrial tissue. *HCN4* changes observed in our study could therefore reflect not only sinus node, but also atrial myocardial changes. However, since HCN4 is highly expressed in the nodal pacemaker cells and only sparsely present in the atrial myocardium (Chandler et al., 2009; Li et al., 2014), it is likely that the *HCN4* changes reflect sinus node, rather than non-nodal *HCN4* alterations. *HCN* changes were only assessed by RNA quantification. Due to the small size of the rat right atrium, additional tissue analyses could not be performed; changes at the protein and *I*_f_ levels were not investigated. Although altered gene expression is expected to result in protein variations, post-transcriptional and post-translational regulatory mechanisms may also influence protein level and function. Diastolic blood pressure was not assessed in the present study. Although diastolic blood pressure changes are also reflected in the SBP, a potential direct impact of ivabradine on the vagal-induced vasodepressor response cannot be excluded. However, given that ivabradine is a pure HCN channels blocker, direct interference at the vascular level is highly unlikely. In the present study, vagal modulation of the HR was evaluated using HRV analysis. Vagal tone evaluation using muscarinic receptors blockade, the Goldberger index, or vagal nerve activity recordings would have also been of interest. Finally, we acknowledge that by causing reduction in baseline HR, ketamine anesthesia could have diminished the impact of vagus nerve stimulation on the HR. However, whereas ketamine anesthesia manifested similar effects on the HR in the ivabradine-treated and non-treated rats, the HR response to vagus nerve stimulation was only abolished in the IVA rats. Thus, the lack of HR response to vagus nerve stimulation observed in the ivabradine-treated rats cannot be ascribed to the effects of ketamine anesthesia.

## Conclusion

The present study demonstrates that long-term ivabradine therapy produces a significant increase in vagal modulation and shifts the sympatho-vagal balance toward vagal dominance. This increase in vagal modulation induced by ivabradine could contribute to the improved outcomes observed in patients with sympathetic hyperactivity, but could also provide an explanation for the increased risk of atrial fibrillation associated with ivabradine therapy in clinical trials. Ivabradine abolished the cardioinhibitory and blunted the hemodynamic response to acute vagal activation in rats, suggesting that *I*_f_ blockade may emerge as a promising therapy for patients with cardioinhibitory vaso-vagal syncope. Our data indicate sinus node *HCN4* up-regulation as a potential mechanism underlying the protective effect of ivabradine against excessive vagal-induced bradycardia in rats.

## Data Availability

The raw data supporting the conclusion of this article will be made available by the authors, without undue reservation.

## Abbreviations

ANS, autonomic nervous system; GAPDH, glyceraldehyde 3-phosphate dehydrogenase; HCN, hyperpolarization-activated cyclic nucleotide-gated channels; HF, high-frequency; HR, heart rate; HRV, heart rate variability; LF, low-frequency; NS, non-significant; pNN5, percentage of successive RR intervals that differ by > 5 ms; RMSSD, root mean square of the successive RR-interval differences; SBP, systolic blood pressure; SDNN, standard deviation of normal RR intervals; SNDR, sinus node discharge rate.
